# Cerebral Micro-Bleeding Detection Based on Densely Connected Neural Network

**DOI:** 10.3389/fnins.2019.00422

**Published:** 2019-05-17

**Authors:** Shuihua Wang, Chaosheng Tang, Junding Sun, Yudong Zhang

**Affiliations:** ^1^School of Computer Science and Technology, Henan Polytechnic University, Jiaozuo, China; ^2^Department of Informatics, University of Leicester, Leicester, United Kingdom

**Keywords:** DenseNet, CMB detection, transfer learning, cost matrix, deep learning

## Abstract

Cerebral micro-bleedings (CMBs) are small chronic brain hemorrhages that have many side effects. For example, CMBs can result in long-term disability, neurologic dysfunction, cognitive impairment and side effects from other medications and treatment. Therefore, it is important and essential to detect CMBs timely and in an early stage for prompt treatment. In this research, because of the limited labeled samples, it is hard to train a classifier to achieve high accuracy. Therefore, we proposed employing Densely connected neural network (DenseNet) as the basic algorithm for transfer learning to detect CMBs. To generate the subsamples for training and test, we used a sliding window to cover the whole original images from left to right and from top to bottom. Based on the central pixel of the subsamples, we could decide the target value. Considering the data imbalance, the cost matrix was also employed. Then, based on the new model, we tested the classification accuracy, and it achieved 97.71%, which provided better performance than the state of art methods.

## Introduction

Cerebral micro-bleeding (CMB) are small chronic brain hemorrhages that can be caused by structural abnormalities of the small vessels of the brain. According to the recent research reports, the causes of CMBs also can be some other common reasons, including high blood pressure, head trauma, aneurysm, blood vessel abnormalities, liver disease, blood or bleeding disorders and brain tumors (Martinez-Ramirez et al., 2014). It also can be caused by some unusual etiologies, such as cocaine abuse, posterior reversible encephalopathy, brain radiation therapy, intravascular lymphomatosis, thrombotic thrombocytopenic purpura, moyamoya disease, infective endocarditis, sickle cell anemia/β-thalassemia, proliferating angio-endotheliomatosis, cerebral autosomal dominant arteriopathy subcortical infarcts, leukoencephalopathy (CADASIL), genetic syndromes, or obstructive sleep apnea (Noorbakhsh-Sabet et al., 2017). The patients suffering from CMBs can have symptoms where the corresponding area that is controlled by the bleeding area malfunctions, resulting in a rise in intracranial pressure due to the large mass putting pressure on the brain and so on. CMBs could be easily ignored as the similar symptoms and signs of the subarachnoid hemorrhages, unless the patients have more obvious symptoms, such as a headache followed by vomiting. Those symptoms can eventually become worse or occur suddenly, based on the distribution and intensity of the CMBs. Patients suffering from CMBs can result in cognitive impairment, neurologic dysfunction and long-term disability. CMBs could also induce side effects from medication or treatments. The worse thing is that the death is possible and can happen quickly. Therefore, the early and prompt diagnosis of CMBs is essential and helpful in timely medical treatment.

Due to the paramagnetic susceptibility of the hemosiderin (Allen et al., 2000), CMBs can be visualized by T2^∗^-gradient recalled echo (GRE) imaging or susceptibility weighted imaging (SWI). Traditionally, CMBs are manually interpreted based on criteria including shapes, diameters and signal characteristics after imaging. However, the criteria were varied as reported in different studies (Cordonnier et al., 2007), until 2009 when Greenberg et al. (2009) published the consensus on standard criteria for CMB identification. However, manual detection methods involve the human interventions, which can bring biases. Meanwhile, the manual detection is labor intensive, hard to reproduce and difficult to exclude the mimics, which can lead to misdiagnosis.

Therefore, the development of automatic CMB detection is important and essential for the accurate detection and early diagnosis of CMBs. Due to the benefits of advanced imaging technologies, massive computer vision aided systems have been developed for automatic CMB detection. For example, Ateeq et al. (2018) proposed a system based on an ensemble classifier. Their system consisted of three steps: first the brain was extracted, then the initial candidates were detected based on the filter and threshold, and finally, feature extraction and classification model were built to remove the false alarms. Fazlollahi et al. (2015) proposed using a multi-scale Laplacian of Gaussian (msLoG) technique to detect the potential CMB candidates, followed by extracting a set of 3-dimensional Radon and Hessian based shape descriptors within each bounding box to train a cascade of binary random forests. Barnes et al. (2011) proposed a statistical thresholding algorithm to recognize the potential hypo-intensities. Then, a supervised classification model based on the support vector machine was employed to distinguish true CMBs from other marked hypo-intensities. van den Heuvel et al. (2016) proposed an automatic detection system for microbleeds in MRIs of patients with trauma based on twelve characteristics related with the dark and spherical characteristics of CMBs and the random forest classifier. Bian et al. (2013) proposed a 2D fast radial symmetry transform (RST) based method to roughly detect the possible CMBs. Then the 3D region growing on the possible CMBs was utilized to exclude the falsely identified CMBs. Ghafaryasl et al. (2012) proposed a computer aided system based on following three steps: skull-stripping, initial candidate selection and reduction of false-positives (FPs) by a two-layer classifier. Zhang et al. (2017) proposed voxel-vise detection based on a single hidden layer feed-forward neural network with scaled conjugate gradient. Chen (2017) proposed a seven-layer deep neural network based on the sparse autoencoder for voxel detection of CMBs. Seghier et al. (2011) proposed a system named MIDAS for automatic CMB detection.

All above methods have reached great progress in CMB detection. However, their detection accuracy and robustness are still in need of improvement.

Therefore, in this paper, we employed the SWI for CMB imaging, which was because SWI could provide high resolution as reported in Haacke et al. (2009) and work as the most sensitive techniques to visualize CMBs. Considering the limited amounts of labeled images, and knowledge to recognize representative characters about the medical images, we considered utilizing the DenseNet as the basic algorithm for transfer learning. The reason for this is because the amount of labeled CMB images is typically very limited, and it is hard to effectively train a classifier to get high detection accuracy. In summary, we proposed using transfer learning of DenseNet for CMB detection based on the collected images, which means we use the knowledges obtained from training the related tasks by DenseNet for CMB detection.

The remainder of this paper is organized in a structure as follows: “Materials and Methods” section describes the method used in this research, “Transfer Learning” section explains why we employed the transfer learning, “CMB Detection Based on the Transfer Learning and DenseNet” section describes the research materials used in this paper, including the training set and test set, and also offers the experiment results, and finally, “Discussion” section provides the conclusion and discussion.

## Materials and Methods

In recent years, Deep Learning (DL) has achieved great progress in object recognition (Tong et al., 2019; Xie et al., 2019), prediction (Yan et al., 2018; Hao et al., 2019), speech analysis (Cummins et al., 2018), noise reduction (Islam et al., 2018), monitoring (Li et al., 2018; Wang et al., 2018), medicine (Raja et al., 2015; Safdar et al., 2018), the recommendation system (Zhang and Liu, 2014), biometrics (Xing et al., 2017) and so on. Traditionally, DL consists of multiple layers of non-linear processing units to obtain the features. The cascaded layers take the output from their previous layer as input. In order to explore the potential of DL, many researchers tried to make the network deeper and wider. However, it suffers from either exploding or the vanishing gradient problem (VGP). Therefore, multiple different structures of DL were proposed. For example, AlexNet, the winner of ImageNet Large Scale Visual Recognition Competition (ILSVRC) 2012, was proposed by Krizhevsky et al. (2012) and has the same structure as LeNet but has max pooling and ReLU non-linearity. VGGNet, proposed by Karen Simonyan (2015), won the second place in ILSVRC 2014 and consisted of deeper networks (19 layers) compared to AlexNet. GoogLeNet, the winner of ILSVRC 2014, provides a deeper and wider network that incorporates 1 × 1 convolutions to reduce the dimensionality of the feature maps before expensive convolutions, together with parallel paths with different receptive field sizes to capture sparse patterns of correlations in the feature map stacks. ResNet, the winner of ILSVRC 2015, offers a 152-layer network that introduces a skip at least two layers or shortcut connections (He et al., 2016). Huang et al. (2017) proposed DenseNet where each layer takes the output from all previous layers in a feed-forward fashion and offers *L*(*L*+1)/2 connections for *L* layers, while traditional convolution networks with *L* layers provide *L* connections. According to the report in Huang et al. (2017), DenseNet can beat the state-of-the-art ResNet structure on ImageNet classification task.

Considering the outstanding performance of DenseNet, we proposed employing DenseNet for cerebral microbleed detection in this paper. The detail of DenseNet was introduced as follows. However, before providing the illustration of the DenseNet, we would first introduce the traditional convolution neural network (CNN) and figure out the difference between CNN and DenseNet later.

### Traditional Convolution Neural Network

**FIGURE 1:**
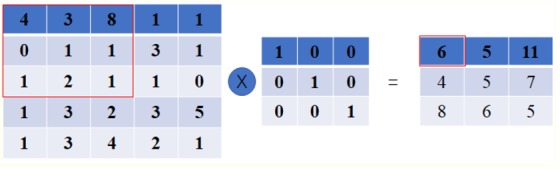
A toy example of convolution operation in CNN with stride size as 1, in which, the left matrix means the input, the second matrix means the kernel, and the right matrix stands for the generated feature map after convolution operation. It is different from the convolution defined in purely mathematic terms.

The traditional CNN usually includes convolution layer, ReLU Layer, pooling layer, fully connected layer and softmax layer (Zeng et al., 2014, 2016a,c, 2017a,b). The functions of different layers are introduced as follows:

Convolution layer works as the core session of a CNN. The feature maps are generated via the convolution of the input with different kernels. Mathematically, it can be expressed as Figure 1, which shows a toy example of convolution operation.

Then, following the convolution layer, we have non-linear activation function, named ReLU, which works to obtain the non-linear features. The purpose of the ReLU layer is to introduce non-linearity into the network. The mathematic expression of ReLU is shown as Eq. 1:

(1) $$\begin{array}{c} f(x)=x^{+}=\max(0,x) \end{array}$$

The pooling layer works by resizing the feature maps spatially to decrease the number of parameters, memory footprint and to make the computation less intensive in the network. The pooling function works on each feature map, the main approaches used for pooling are max pooling as Eq. 2, average pooling as Eq. 3:

(2) $$\begin{array}{c} a_{j}=\underset{i\in R_{j}}{\max}(M_{i}) \end{array}$$

(3) $$\begin{array}{c} a_{j}=\frac{1}{\left|R_{j}\right|}\sum_{i\in R_{j}}M_{i} \end{array}$$

In which *M* stands for the pooling region and *Rj* represents for the number of elements within the pooling region.

Fully connected layers will calculate the confidential scores, which are stored in a volume of size 1 × 1 × *n*. Here, *n* means the number of categories, and each element stands for class scores. Every neuron of the fully connected layer is connected to all the neurons in the earlier layers.

### Structure Revision of the CNN

In the traditional CNN, all layers are connected gradually as in Eq. 4:

(4) $$\begin{array}{c} x_{l}{=H}_{l}(x_{l-1}) \end{array}$$

However, as the network becomes deeper and wider, the networks may suffer from either exploding or gradient vanishing. Therefore, researchers proposed different network structures to overcome this problem. For example, ResNet revised this behavior by short connection, and the equation is reformulated as (5).

(5) $$\begin{array}{c} x_{l}{=H}_{l}(x_{l-1})+x_{l-1} \end{array}$$

Instead of making the sum of the output feature maps of the layer with the incoming feature maps, DenseNet concatenates them sequentially. The expression is reformulated into Eq. 6:

(6) $$\begin{array}{c} x_{l}=H_{l}([x_{0},x_{l},x_{2},...,x_{l-1}]) \end{array}$$

In which *l* means the index of the layer number, *H* stands for a non-linear operation and *x_l_* stands for the output of the *l*th layer.

### DenseNet

**FIGURE 2:**
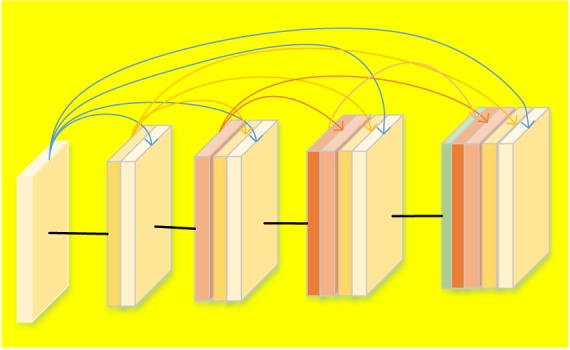
Structure of the DenseBlock (5 layers and each layer takes feature maps from all previous layers).

As expressed in Eq. 6, DenseNet introduces straight forward connections from any layers to all following layers. In other words, the *l*th layer receives feature-maps from all previous *l* – 1 layers. However, if the feature maps’ size changes, the concatenation operation is not feasible. Therefore, down-sampling to change the size of the feature maps are introduced. In order to make the down-sampling in the structure of DenseNet possible, multiple densely connected dense blocks are introduced to divide the network. The layers between the blocks are named as transition layers that have batch normalization, convolution and pooling operations, as shown in Figure 2. Figure 2 describes a case of DenseBlock, in which the layer number is 5 and the growth rate is set as *k*. Each layer receives feature maps from all earlier layers.

For each operation H_l_, it generates *k* feature maps, which is defined as growth rate. Therefore, the l_th_ layer will have k_0_ + k(l - 1) feature maps, and k_0_ is the number of channels in the input layer. As the network typically has a large number of inputs, a 1 × 1 convolution is employed as the bottleneck layer before the 3 × 3 convolution layer to reduce the feature maps and improve the computation efficiency.

**FIGURE 3:**
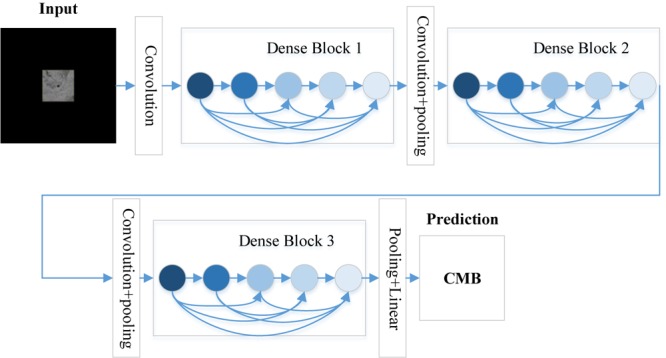
The structure of the DenseNet.

To further compress the model to improve the model compactness, the feature maps are further reduced by the transition layer. For example, if a dense block generates *m* feature maps and the compression factor is set as 𝜃 ∈ (0,1], then the feature maps will be reduced to ⌊𝜃*m*⌋ via the followed transition layer. If 𝜃 = 1, the number of feature maps will be the same. Figure 3 shows the structure of DenseNet, which is composed of three DenseBlocks, an input layer and transition layers. The cropped samples are used as the input, the final layer will tell us whether it is CMB or Non-CMB in this research.

## Transfer Learning

DenseNet has been widely applied in the medical research. For example, Gottapu and Dagli (2018) proposed using DenseNet for Anatomical Brain Segmentation. Khened et al. (2019) proposed cardiac segmentation based on fully convolutional multi-scale residual DenseNets. Wang H. et al. (2019) offered a system for recognition of mild cognitive impairment (MCI) and Alzheimer’s disease (AD), based on the ensemble of 3D densely connected convolution network. Considering the limited amounts of labeled training samples, it is far way from enough to retrain the whole network of DenseNet from scratch to get a high classification accuracy. Therefore, in this paper, we proposed transfer learning, which means frozen the earlier layers and retrain the later layers of DenseNet for CMB detection task. The structure of DenseNet used here is DenseNet 201.

In order to make the pretrained DenseNet 201 for CMB detection feasible, which was a binary classification of CMB or non-CMB, the fully connected (FC) layer with 1000 neuron was replaced by a new FC layer with 2 neurons. The structure of the remaining part of DenseNet 201 was unchanged.

## CMB Detection Based on the Transfer Learning and Densenet

### Materials

The subjects used in this research are ten healthy controls and ten patients of CADASIL. Twenty 3D volumetric images were obtained from the 20 patients. Then, Software Sygno MR B17 was utilized to rebuild the 3D volumetric image. Each 3D volumetric image’s size is uniformly set as 364^∗^448^∗^48.

In order to mark the CMBs from the subjects manually, we employed three neuron-radiologists with more than twenty-years’ experience. The rules were set as follows: (1) via tracking the neighbor slices, blood vessels were first excluded, (2) lesions should be smaller than 10 mm in diameter. The potential CMBs were labeled as either “possible” or “Definite,” Otherwise, regarded as non-CMB voxels. In case of the conflictions, we proposed to obey the rule that the minority should be subordinate to the majority.

The sample images were generated from the original image. We applied the sliding window whose size is set as 61 by 61 to the original image. The border pixels were discarded due to the fat and brain skull. All the pixels located within a sliding window were used as one input, and the point located in the center of the sliding window was used as the target value. It means that if the central pixel is true or 1, then the target value is 1, otherwise, the target label is set as 0. It is expressed in the Eqs 7 and 8:

(7) $$\begin{array}{c} I=W(p) \end{array}$$

(8) $$\begin{array}{c} Ou=\{\begin{array}{l} 1,\;\mathrm{Central}\;\mathrm{pixel}\;p\;\mathrm{is}\;\mathrm{true}\;(\mathrm{CMB})\\ 0,\;\mathrm{Central}\;\mathrm{pixel}\;p\;\mathrm{is}\;\mathrm{false}\;(non-CMB) \end{array} \end{array}$$

**FIGURE 4:**
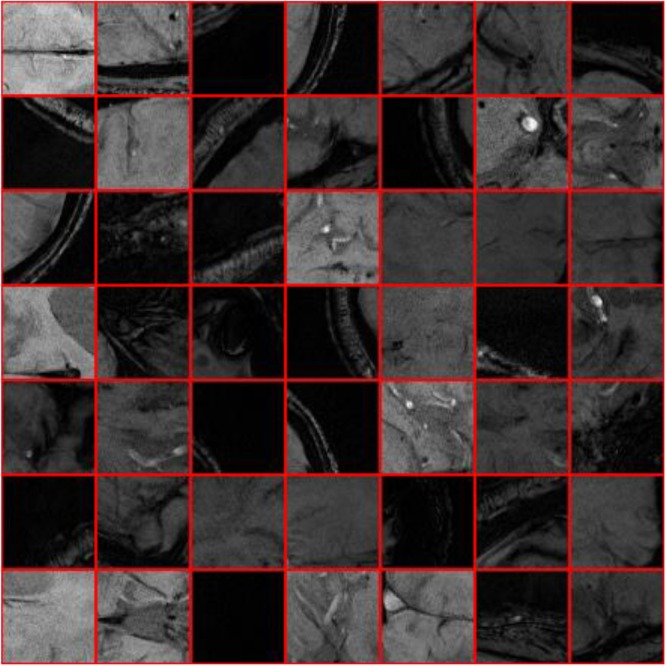
Non-CMB samples.

Where *I* stands for the cropped sample images generated via the sliding window, *p* represents for the central pixel, *W*(*p*) means the pixels which centered on pixel *p* and were located inside the sliding window, and *Ou* means the label value. Figure 4, 5 show the sample of CMB and non-CMB centered images.

**FIGURE 5:**
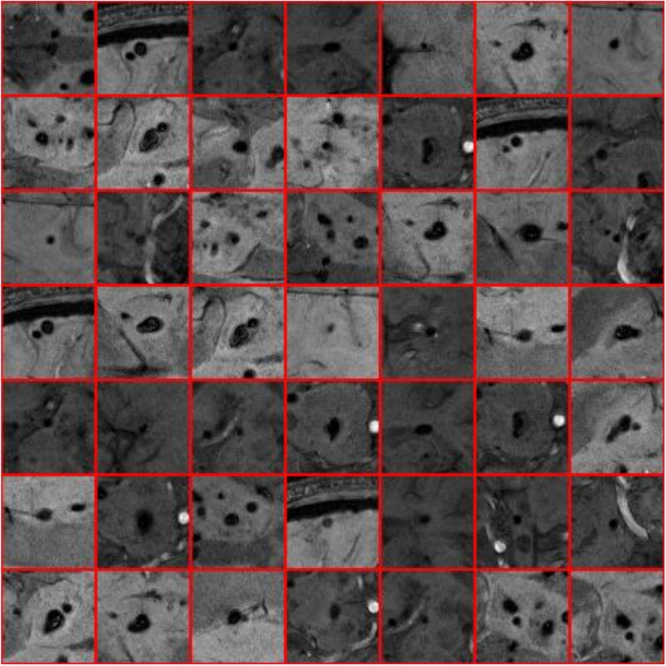
CMB samples.

The sliding window was supposed to cover the image from left to right and top to bottom with the stride size as 1. Therefore, we got the total CMB voxels as 68, 847 and non-CMB voxels as 56, 582, 536. The training and test set was divided as Table 1. We randomly selected 10000 images for each category of the test, and the remaining images were used for training.

**Table 1:** Dividing of the dataset for training and testing.

	Train	Test
CMB	58, 847	10000
Non-CMB	56, 572, 536	10000

**FIGURE 6:**
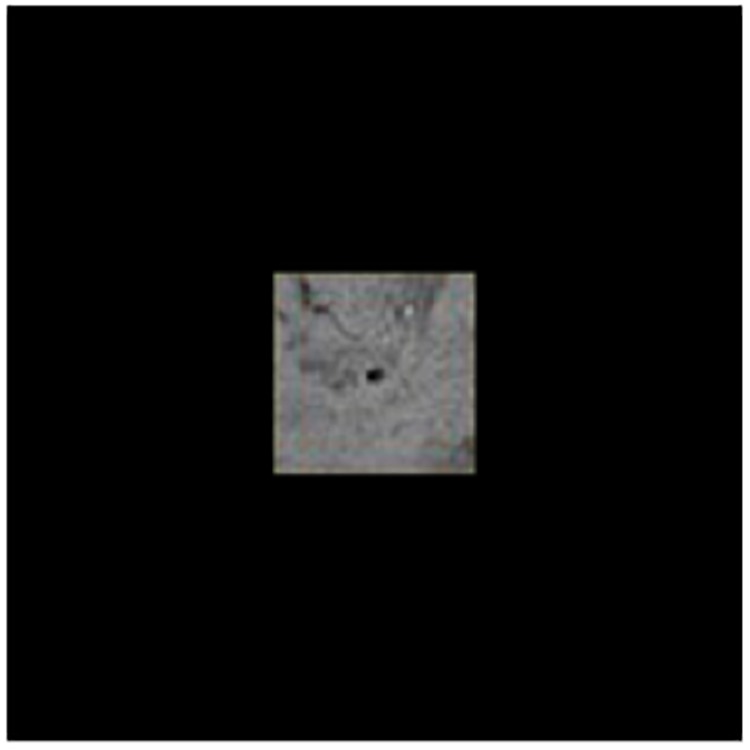
Images padded for DenseNet.

**FIGURE 7:**
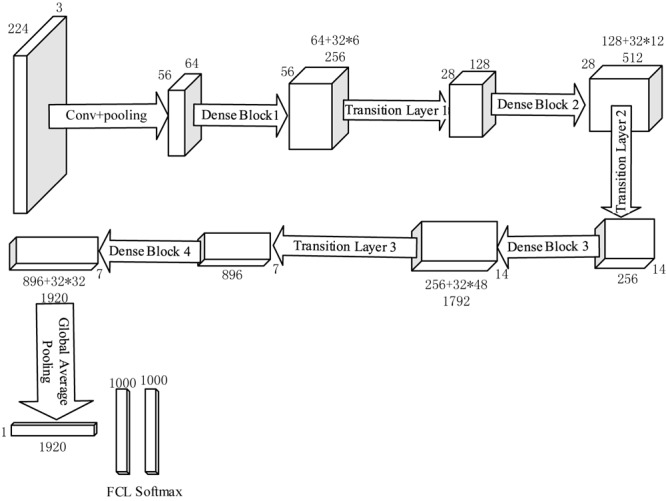
Flowchart of DenseNet 201.

To make the images suitable for DenseNet, which should be resized as 224 × 224 × 3, we padded the images with zero. The preprocessed image sample is shown as Figure 6. Then Figure 7 shows the flowchart of the DenseNet, including number of feature maps generated by each layer.

From Table 1, we can find that the Non-CMB training data dominates the majority type CMB, which will cause the classifier more bias toward to the Non-CMB. Therefore, it may cause difficulties in controlling false positives and false negatives, which means the model is hard to find the CMB samples. Therefore, in order to overcome this challenge, we introduced cost matrix (Wu and Kim, 2010). The cost ratio ct was set as 961 via Eq. 9:

(9) $$\begin{array}{c} ct=N_{non-CMB}/N_{CMB} \end{array}$$

In which N_non-CMB_ means the number of non-CMB training samples and N_CMB_ stands for number of CMB training samples. The reason for why we employ the cost matrix instead of over sampling or down sampling is mainly because we have more concerns about the false positives and false negatives, therefore it is better to highlight the imbalanced learning problem by using cost matrices instead of creating a balanced data distribution forcefully.

### Experiment Design

The goal of this research is to identify the input image as either CMB or Non-CMB. In order to achieve this goal, we proposed using DenseNet 201 as the basic algorithm for transfer learning, based on the excellent performance of DenseNet on ImageNet classification task. Section “Materials” stated the materials used in this research. Based on the original images, we created 68, 847 CMB subsamples and 56, 582, 536 Non-CMB subsamples. 10000 samples were randomly selected as test samples. The remaining sub-samples were used for training. In order to overcome the problem of data imbalance, we proposed cost matrix to show the more concerns in false positive and false negatives. The experiment is carried on by Matlab on the Windows 10 Operation System with 2.88 GHz processor and 8 GB memory. The following experiments were carried out: (1) CMB detection based on DenseNet. The measurements used here include accuracy, sensitivity and specificity. The definition of the measurements can be found in Zhang et al. (2018b). (2) Different cutting points of transfer learning. (3) In order to show the performance of proposed methods, we compared with other state of art work. Considering the measurements provided in other research, we only used sensitivity, specificity and accuracy.

In order to provide better illustration of DenseNet, we added a flowchart with feature map size, learnable weights of each layer. As we only noted the size of the width, the length should be same with the width.

### CMB Detection Result Based on DenseNet

**FIGURE 8:**
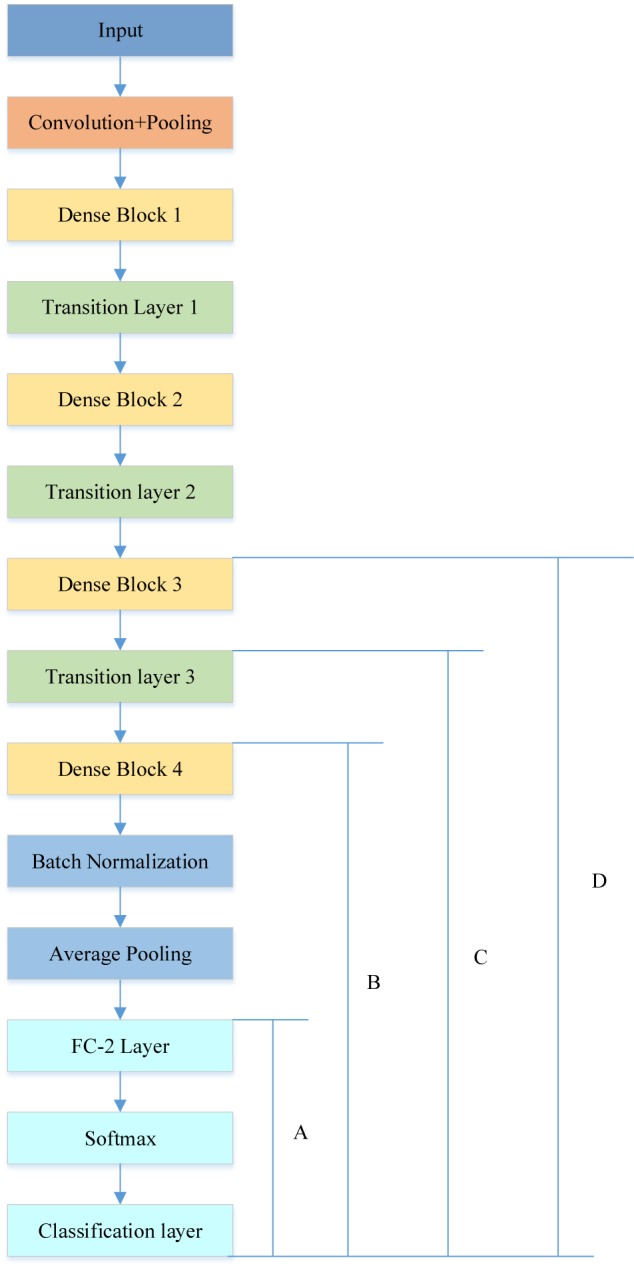
Different cases of transfer learning (the original fully connected layer with 1000 neurons was replaced by a new fully connected layer with 2 neurons).

The rebuilt network was composed of four DenseBlocks, one input layer, three transition layers, one fully connected layer with two neurons, a softmax layer and a classification layer, as described in Figure 8.

Table 2 provides the detection result. The correctly detected CMBs were 9777, and for Non-CMB they were 9764. 236 non-CMBs were incorrectly detected as CMBs, and 223 CMBs were wrongly detected as non-CMBs. The sensitivity was achieved as 97.78%, the specificity was 97.64%, the accuracy was 97.71% and the precision was 97.65%. Above measurements were obtained based on the average of 10 runs as shown in Table 3.

**Table 2:** Confusion matrix of detected CMB and Non-CMB.

	Predicted	
Actual	CMBs	Non-CMBs
CMBs (10000)	9777	223
Non-CMBs (10000)	236	9764

**Table 3:** Measurements value CMB detection based on transfer learning of DenseNet (Units: %).

Measurements	Sensitivity	Specificity	Accuracy	Precision
R 1	96.69	96.73	96.71	96.72
R 2	97.82	97.87	97.84	97.90
R 3	98.71	98.51	98.61	98.52
R 4	96.71	96.27	96.49	96.29
R 5	96.69	96.19	96.44	96.22
R 6	96.93	96.99	96.96	97.00
R 7	98.44	98.40	98.42	98.39
R 8	98.77	98.58	98.67	98.58
R 9	98.71	98.62	98.67	98.62
R 10	98.30	98.22	98.26	98.22
	97.78 ± 0.88	97.64 ± 0.94	97.71 ± 0.90	97.65 ± 0.93

### Comparison to the Different Cases of Transfer Learning

In order to achieve the best performance of transfer learning, different cutting points for transfer learning were designed as shown in Figure 8. Due to the limited subjects, we mainly focused on retraining the later layers of DenseNet. Therefore, in case A, the DenseNet 201 except for the last three layers, was used as the feature extractor for this research, and we retrained the newly added three layers.

In case B, C, and D, we included extra layers for retraining. For example, case B retrained the DenseBlock 4, Batch normalization, Average pooling, Fully connected (FC) layer, softmax layer and classification layer. It was implemented via setting the learning rate to 0 for earlier layers and setting learning rate factor to 10 for layers to be retrained. Table 4 illustrates the comparison results.

**Table 4:** Comparison of different cases of transfer learning (Unit: %).

	Sensitivity	Specificity	Accuracy	Precision
Case A	97.78 ± 0.88	97.64 ± 0.94	97.71 ± 0.90	97.65 ± 0.93
Case B	97.56 ± 0.83	97.65 ± 0.76	97.60 ± 0.79	97.67 ± 0.76
Case C	97.36 ± 1.05	97.66 ± 0.8	97.51 ± 0.92	97.66 ± 0.82
Case D	97.61 ± 0.63	97.54 ± 0.65	97.58 ± 0.64	97.57 ± 0.65

**FIGURE 9:**
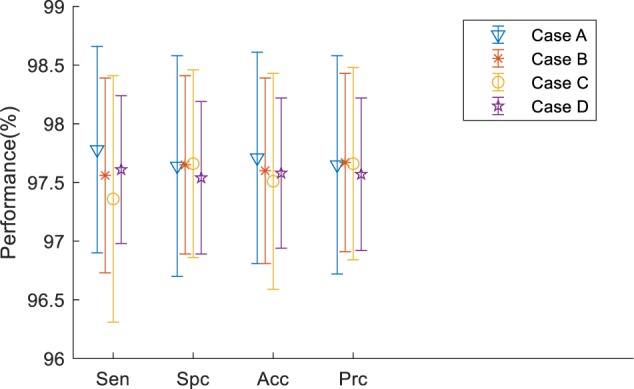
Error bar.

From Table 4, we can find that Case A performed slightly better than the other three cases in the terms of sensitivity and accuracy. Considering that in medical research, we focus more on the sensitivity and accuracy than on the other two terms, we thought Case A provided the best performance among all the cases. Figure 9 shows the error bar of the measurement values. From the point of storage consuming, all four cases take about the same RAM as we did when not using the precomputation method.

### Comparison to the State of Art Work

In order to validate our proposed method, we compared different state of the art methods, including traditional machine learning methods and DL methods.

From Table 5, we compared our method with single-hidden layer feed-forward neural-network (SLFN)+ (leaky) rectified linear unit, 4-layer sparse encoder, 7-layer sparse encoder, different layers of CNN, Naive Bayesian Classifier and so on. We can find that our proposed method offers the best performance. DenseNet works as a logical extension of ResNet but provides more compact models and fully uses the features.

**Table 5:** Comparison to the state of art methods.

Method	Sensitivity	Specificity	Accuracy
SNP+SLFN+LReLU (Zhang et al., 2018c)	93.05	93.06	93.06
4-layer SAE (Zhang et al., 2016)	93.20 ± 1.37	93.25 ± 1.38	93.22 ± 1.37
7-layer SAE (Zhang et al., 2018d)	95.13 ± 0.84	93.33 ± 0.84	94.23 ± 0.84
CNN + RAP (Wang et al., 2017)	96.94	97.18	97.18
CNN (Lu et al., 2017)	97.29	92.23	96.05
NBC (Bao et al., 2018)	74.53 ± 0.96	74.51 ± 1.05	74.52 ± 1.00
GA-BPNN (Cloutie, 2018)	72.90 ± 1.38	72.89 ± 1.18	72.90 ± 1.28
CNN-SP (Wang S. et al., 2019)	97.22	97.35	97.28
Our method	97.78 ± 0.88	97.64 ± 0.94	97.71 ± 0.90

**FIGURE 10:**
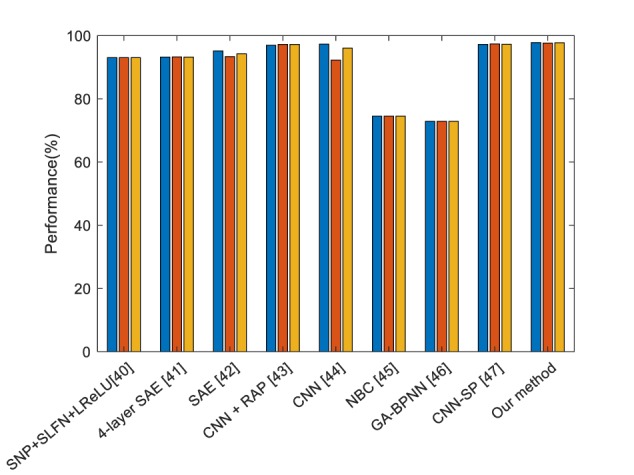
Comparison of the state of art methods (Blue means the sensitivity, red means the specificity, and yellow means the accuracy).

Figure 10 shows the bar chart of the comparison of the state of the state of art methods. It shows that our proposed method performs slightly better than the current best method, but largely improved compared to the traditional method naïve Bayes classifier (NBC).

## Discussion

In this paper, we proposed to employ DenseNet to detect CMBs in patients with CADASIL. DenseNet was proposed by Huang et al. (2017) and competed the other DL methods for ImageNet classification task because of its model compactness and fully used features. DenseNet are quite similar with ResNet, however, instead of the summation, DenseNet proposed the concatenation of all feature maps from previous layers, which encourages the feature reuse, the VGP alleviation, and the decreased number of parameters.

Therefore, in this paper, we proposed using DenseNet for CMB detection by supposing CMB detection has similarity with ImageNet classification. However, because of the data imbalance, we utilized cost matrix to avoid the model bias toward non-CMB, which means the model would be hard to find CMBs if trained under the imbalanced dataset. As there are some other methods for data imbalance, such as over sampling and down sampling, we have more concerns about the false negatives or false positives. Therefore, instead of enforcing the data into balanced distribution, we employed the cost matrix. In order to check the best cutting point, we test different cases of transfer learning and the results are shown in Table 4, however, the difference is not so obvious. On the other hand, training less layers can help us save time and decrease the computation cost if we import the strategy of precomputation.

In the future, we will try to collect more samples and test more different structures for CMB detection. Meanwhile, the training cost long term is very high (Zeng et al., 2018), therefore it is necessary to optimize the algorithm to make the training fast (Zeng et al., 2016b,d). We will consider other precomputation and some optimization methods (Zhang et al., 2018a).

## Data Availability

The datasets for this manuscript are not publicly available because due to the privacy of the subjects. Requests to access the datasets should be directed to shuihuawang@ieee.org.

## Ethics Statement

This research was approved by Institutional Review Board (IRB) of the First Affiliated Hospital of Nanjing Medical University. We obtained written informed consent from the participants of this study.

## Author Contributions

SW proposed the study and wrote the draft. CT and JS designed the model and interpreted the results. CT and YZ analyzed the data. SW and YZ acquired the preprocessed data. All authors gave critical revision and consent for this submission.

## Conflict of Interest Statement

The authors declare that the research was conducted in the absence of any commercial or financial relationships that could be construed as a potential conflict of interest.

## References

[B1] Allen P. D. St Pierre T. G. Chua-anusorn W. Ström V. Rao K. V. (2000). Low-frequency low-field magnetic susceptibility of ferritin and hemosiderin. *Biochim. Biophys. Acta* 1500 186–196. 10.1016/s0925-4439(99)00104-0 10657588

[B2] Ateeq T. Majeed M. N. Anwar S. M. Maqsood M. Rehman Z.-U. Lee J. W. (2018). Ensemble-classifiers-assisted detection of cerebral microbleeds in brain MRI. *Comput. Electr. Eng.* 69 768–781. 10.1016/j.compeleceng.2018.02.021

[B3] Bao F. Shen M. Macdonald F. (2018). Voxelwise detection of cerebral microbleed in CADASIL patients by naïve Bayesian classifier. *Adv. Intel. Syst. Res.* 148 176–180.

[B4] Barnes S. R. S. Haacke E. M. Ayaz M. Boikov A. S. Kirsch W. Kido D. (2011). Semiautomated detection of cerebral microbleeds in magnetic resonance images. *Mag. Reson. Imag.* 29 844–852. 10.1016/j.mri.2011.02.028 21571479 PMC3118856

[B5] Bian W. Hess C. P. Chang S. M. Nelson S. J. Lupo J. M. (2013). Computer-aided detection of radiation-induced cerebral microbleeds on susceptibility-weighted MR images. *NeuroImage Clin.* 2 282–290. 10.1016/j.nicl.2013.01.012 24179783 PMC3777794

[B6] Chen H. (2017). Seven-layer deep neural network based on sparse autoencoder for voxelwise detection of cerebral microbleed. *Multimedi. Tools Appl.* 77 10521–10538. 10.1007/s11042-017-4554-8

[B7] Cloutie R. S. (2018). Voxelwise detection of cerebral microbleed in CADASIL patients by genetic algorithm and back propagation neural network. *Adv. Comput. Sci. Res.* 65 101–105.

[B8] Cordonnier C. Al-Shahi Salman R. Wardlaw J. (2007). Spontaneous brain microbleeds: systematic review, subgroup analyses and standards for study design and reporting. *Brain* 130 1988–2003. 10.1093/brain/awl387 17322562

[B9] Cummins N. Baird A. Schuller B. W. (2018). Speech analysis for health: current state-of-the-art and the increasing impact of deep learning. *Methods* 151 41–54. 10.1016/j.ymeth.2018.07.007 30099083

[B10] Fazlollahi A. Meriaudeau F. Giancardo L. Villemagne V. L. Rowe C. C. Yates P. (2015). Computer-aided detection of cerebral microbleeds in susceptibility-weighted imaging. *Comput. Med. Imag. Graph.* 46 269–276. 10.1016/j.compmedimag.2015.10.001 26560677

[B11] Ghafaryasl B. Lijn F. V. D. Poels M. Vrooman H. Ikram M. A. Niessen W. J. (2012). “A computer aided detection system for cerebral microbleeds in brain MRI,” in *Proceedings of the 2012 9th IEEE International Symposium on Biomedical Imaging (ISBI)* Barcelona 138–141.

[B12] Gottapu R. D. Dagli C. H. (2018). DenseNet for anatomical brain segmentation. *Proc. Comput. Sci.* 140 179–185. 10.1016/j.compmedimag.2018.09.009 30340094

[B13] Greenberg S. M. Vernooij M. W. Cordonnier C. Viswanathan A. Al-Shahi Salman R. Warach S. (2009). Cerebral microbleeds: a guide to detection and interpretation. *Lancet Neurol.* 8 165–174. 10.1016/S1474-4422(09)70013-4 19161908 PMC3414436

[B14] Haacke E. M. Mittal S. Wu Z. Neelavalli J. Cheng Y.-C. N. (2009). Susceptibility-weighted imaging: technical aspects and clinical applications, part 1. *Am. J. Neuroradiol.* 30 19–30. 10.3174/ajnr.a1400 19039041 PMC3805391

[B15] Hao Y. X. Usama M. Yang J. Hossain M. S. Ghoneim A. (2019). Recurrent convolutional neural network based multimodal disease risk prediction. *Fut. Gener. Comput. Syst.* 92 76–83. 10.1016/j.future.2018.09.031

[B16] He K. Zhang X. Ren S. Sun J. (2016). “Deep Residual Learning for Image Recognition,” in *Proceedings of the 2016 IEEE Conference on Computer Vision and Pattern Recognition (CVPR)* (Las Vegas, NV: IEEE).

[B17] Huang G. Liu Z. Maaten L. V. D. Weinberger K. Q. (2017). “Densely Connected Convolutional Networks,” in *Proceedings of the 2017 IEEE Conference on Computer Vision and Pattern Recognition (CVPR)* (Las Vegas, NV: IEEE) 2261–2269.

[B18] Islam M. T. Mahbubur Rahman S. M. Omair Ahmad M. Swamy M. N. S. (2018). Mixed gaussian-impulse noise reduction from images using convolutional neural network. *Signal Process. Image Commun.* 68 26–41. 10.1016/j.image.2018.06.016

[B19] Karen Simonyan Z. (2015). “Very Deep Convolutional Networks for Large-Scale Image Recognition,” in *Proceedings of the Conference Paper at ICLR* New Orleans.

[B20] Khened M. Kollerathu V. A. Krishnamurthi G. (2019). Fully convolutional multi-scale residual DenseNets for cardiac segmentation and automated cardiac diagnosis using ensemble of classifiers. *Med. Image Anal.* 51 21–45. 10.1016/j.media.2018.10.004 30390512

[B21] Krizhevsky A. Sutskever I. Hinton G. E. (2012). “ImageNet classification with deep convolutional neural networks,” in *Proceedings of the 25th International Conference on Neural Information Processing Systems* Lake Tahoe.

[B22] Li S. Sun W. Zhang Y. Liu H. (2018). Reliability analysis for multipath communications in mobile cloud computing architectures. *Wirel. Commun. Mob. Comput.* 2018:8539307.

[B23] Lu S. Lu Z. Hou X. Cheng H. Wang S. (2017). “Detection of cerebral microbleeding based on deep convolutional neural network,” in *Proceedings of the 14th International Computer Conference on Wavelet Active Media Technology and Information Processing (ICCWAMTIP)* Chengdu 93–96.

[B24] Martinez-Ramirez S. Greenberg S. M. Viswanathan A. (2014). Cerebral microbleeds: overview and implications in cognitive impairment. *Alzheimer’s Res. Ther.* 6:33. 10.1186/alzrt263 24987468 PMC4075149

[B25] Noorbakhsh-Sabet N. Pulakanti V. C. Zand R. (2017). Uncommon causes of cerebral microbleeds. *J. Stroke Cerebrovasc. Dis.* 26 2043–2049. 10.1016/j.jstrokecerebrovasdis.2017.07.012 28826581

[B26] Raja K. B. Raghavendra R. Vemuri V. K. Busch C. (2015). Smartphone based visible iris recognition using deep sparse filtering. *Patt. Recogn. Lett.* 57 33–42. 10.1016/j.patrec.2014.09.006

[B27] Safdar S. Zafar S. Zafar N. Khan N. F. (2018). Machine learning based decision support systems (DSS) for heart disease diagnosis: a review. *Artific. Intel. Rev.* 50 597–623. 10.1007/s10462-017-9552-8

[B28] Seghier L. Kolanko M. A. Leff A. P. Jäger H. R. Gregoire S. M. Werring D. J. (2011). Microbleed detection using automated segmentation (MIDAS): a new method applicable to standard clinical MR images. *PLoS One* 6:e17547. 10.1371/journal.pone.0017547 21448456 PMC3063172

[B29] Tong S. Fu Y. Ling H. (2019). Gait recognition with cross-domain transfer networks. *J. Syst. Arch.* 93 40–47. 10.1016/j.sysarc.2019.01.002

[B30] van den Heuvel T. L. van der Eerden A. W. Manniesing R. Ghafoorian M. Tan T. Andriessen T. M. (2016). Automated detection of cerebral microbleeds in patients with traumatic brain injury. *NeuroImage Clin.* 12 241–251. 10.1016/j.nicl.2016.07.002 27489772 PMC4950582

[B31] Wang H. Shen Y. Wang S. Xiao T. Deng L. Wang X. (2019). Ensemble of 3D densely connected convolutional network for diagnosis of mild cognitive impairment and Alzheimer’s disease. *Neurocomputing* 333 145–156. 10.1016/j.neucom.2018.12.018

[B32] Wang S. Jiang Y. Hou X. Cheng H. Du S. (2017). “Cerebral Micro-Bleed Detection Based on the Convolution Neural Network With Rank Based Average Pooling,” in *Proceedings of the IEEE Access* Chengdu 16576–16583. 10.1109/access.2017.2736558

[B33] Wang S. Liu L. Qu L. Yu C. Sun Y. Gao F. (2018). Accurate Ulva prolifera regions extraction of UAV images with superpixel and CNNs for ocean environment monitoring. *Neurocomputing* (in press).

[B34] Wang S. Sun J. Mehmood I. Pan C. Chen Y. Zhang Y.-D. (2019). Cerebral micro-bleeding identification based on nine-layer convolutional neural network with stochastic pooling. *Concurr. Comput. Pract. Exp.* e5130 10.1002/cpe.5130

[B35] Wu M. Kim C. (2010). A cost matrix agent for shortest path routing in ad hoc networks. *J. Netw. Comput. Appl.* 33 646–652. 10.1016/j.jnca.2010.03.013

[B36] Xie C. Li C. Zhang B. Pan L. Ye Q. Chen W. (2019). Hierarchical residual stochastic networks for time series recognition. *Inform. Sci.* 471 52–63. 10.1016/j.ins.2018.08.065

[B37] Xing J. H. Li K. Hu W. M. Yuan C. F. Ling H. B. (2017). Diagnosing deep learning models for high accuracy age estimation from a single image. *Patt. Recogn.* 66 106–116. 10.1016/j.patcog.2017.01.005

[B38] Yan M. Li M. He H. Peng J. (2018). Deep learning for vehicle speed prediction. *Energy Proc.* 152 618–623. 10.1016/j.egypro.2018.09.220

[B39] Zeng N. Y. Wang Z. D. Zhang H. (2016a). Inferring nonlinear lateral flow immunoassay state-space models via an unscented Kalman filter. *Sci. China Inform. Sci.* 59:10.

[B40] Zeng N. Y. Wang Z. D. Zhang H. Alsaadi F. E. (2016b). A novel switching delayed PSO algorithm for estimating unknown parameters of lateral flow immunoassay. *Cogn. Comput.* 8 143–152. 10.1007/s12559-016-9396-6

[B41] Zeng N. Y. Wang Z. D. Zhang H. Liu W. B. Alsaadi F. E. (2016c). Deep belief networks for quantitative analysis of a gold immunochromatographic strip. *Cogn. Comput.* 8 684–692. 10.1007/s12559-016-9404-x

[B42] Zeng N. Y. Zhang H. Chen Y. P. Chen B. Q. Liu Y. R. (2016d). Path planning for intelligent robot based on switching local evolutionary PSO algorithm. *Assemb. Automat.* 36 120–126. 10.1108/aa-10-2015-079

[B43] Zeng N. Y. Wang Z. D. Zineddin B. Li Y. R. Du M. Xiao L. (2014). Image-based quantitative analysis of gold immunochromatographic strip via cellular neural network approach. *IEEE Trans. Med. Imag.* 33 1129–1136. 10.1109/TMI.2014.2305394 24770917

[B44] Zeng N. Y. You Y. Xie L. S. Zhang H. Ye L. S. Hong W. X. (2018). A new imaged-based quantitative reader for the gold immunochromatographic assay. *Optik* 152 92–99. 10.1016/j.ijleo.2017.09.109

[B45] Zeng N. Y. Zhang H. Li Y. R. Liang J. L. Dobaie A. M. (2017a). Denoising and deblurring gold immunochromatographic strip images via gradient projection algorithms. *Neurocomputing* 247 165–172. 10.1016/j.neucom.2017.03.056

[B46] Zeng N. Y. Zhang H. Liu W. B. Liang J. L. Alsaadi F. E. (2017b). A switching delayed PSO optimized extreme learning machine for short-term load forecasting. *Neurocomputing* 240 175–182. 10.1016/j.neucom.2017.01.090

[B47] Zhang Y. Liu H. (2014). DCFRS: A cloud-based distributed collaborative filtering recommender system for mobile users. *ICIC Expr. Lett.* 8 1889–1895.

[B48] Zhang Y. Su Y. Weigang L. Liu H. (2018a). Rumor and authoritative information propagation model considering super spreading in complex social networks. *Phys. A Stat. Mech. Appl.* 506 395–411. 10.1016/j.physa.2018.04.082

[B49] Zhang Y. Wang S. Sui Y. Yang M. Liu B. Cheng H. (2018b). Multivariate approach for alzheimer’s disease detection using stationary wavelet entropy and predator-prey particle swarm optimization. *J. Alzheimers Dis.* 65 855–869. 10.3233/JAD-170069 28731432

[B50] Zhang Y. D. Hou X. X. Chen Y. Chen H. Yang M. Yang J. (2018c). Voxelwise detection of cerebral microbleed in CADASIL patients by leaky rectified linear unit and early stopping. *Multimed. Tools Appl.* 77 21825–21845. 10.1007/s11042-017-4383-9

[B51] Zhang Y. D. Zhang Y. Hou X. X. Chen H. (2018d). Seven-layer deep neural network based on sparse autoencoder for voxelwise detection of cerebral microbleed. *Multimed. Tools Appl.* 77 10521–10538. 10.1007/s11042-017-4554-8

[B52] Zhang Y. D. Hou X. X. Chen Y. Chen H. Yang M. Yang J. (2017). Voxelwise detection of cerebral microbleed in CADASIL patients by leaky rectified linear unit and early stopping^∗^. *Multimed. Tools Appl.* 77 1–21.

[B53] Zhang Y. D. Hou X. X. Lv Y. D. Chen H. Zhang Y. Wang S. H. (2016). “Sparse Autoencoder based deep neural network for voxelwise detection of cerebral microbleed,” in *Proceedings of the 22nd International Conference on Parallel and Distributed Systems^∗^* Wuhan 1229–1232.

